# Cost-effectiveness of rotavirus vaccination in Ghana: Examining impacts from 2012 to 2031

**DOI:** 10.1016/j.vaccine.2017.11.080

**Published:** 2018-11-12

**Authors:** Justice Nonvignon, Deborah Atherly, Clint Pecenka, Moses Aikins, Lauren Gazley, Devin Groman, Clement T. Narh, George Armah

**Affiliations:** a School of Public Health, University of Ghana, Legon, Ghana; b PATH, Seattle, United States; c School of Public Health, University of Health and Allied Sciences, Hohoe, Ghana; d Noguchi Memorial Institute for Medical Research, University of Ghana, Legon, Ghana

**Keywords:** Cost-effectiveness, Rotavirus, Vaccination, Gavi transition, Ghana

## Abstract

**Background:**

Diarrhea causes about 10% of all deaths in children under five years globally, with rotavirus causing about 40% of all diarrhea deaths. Ghana introduced rotavirus vaccination as part of routine immunization in 2012 and it has been shown to be effective in reducing disease burden in children under five years. Ghana’s transition from low to lower-middle income status in 2010 implies fewer resources from Gavi as well as other major global financing mechanisms. Ghana will soon bear the full cost of vaccines. The aim of this study was to estimate the health impact, costs and cost-effectiveness of rotavirus vaccination in Ghana from introduction and beyond the Gavi transition.

**Methods:**

The TRIVAC model is used to estimate costs and effects of rotavirus vaccination from 2012 through 2031. Model inputs include demographics, disease burden, health system structure, health care utilization and costs as well as vaccine cost, coverage, and efficacy. Model inputs came from local data, the international literature and expert consultation. Costs were examined from the health system and societal perspectives.

**Results:**

The results show that continued rotavirus vaccination could avert more than 2.2 million cases and 8900 deaths while saving US$6 to US$9 million in costs over a 20-year period. The net cost of vaccination program is approximately US$60 million over the same period. The societal cost per DALY averted is US$238 to US$332 with cost per case averted ranging from US$27 to US$38. The cost per death averted is approximately US$7000.

**Conclusion:**

The analysis shows that continued rotavirus vaccination will be highly cost-effective, even for the period during which Ghana will assume responsibility for purchasing vaccines after transition from Gavi support.

## Highlights

• Ghana is currently transitioning away from Gavi support.
• Thus, cost-effectiveness is crucial for improving health system efficiency.
• Rotavirus vaccination brings health and economic benefits to Ghana.
• Rotavirus vaccination is highly cost-effective in Ghana, even Gavi transition.

## 1 Introduction

Diarrhea is a major public health challenge, accounting for nearly 10% of all deaths in children under five years globally [1], [2]. Rotavirus is the leading cause of diarrheal deaths, being responsible for approximately 40% of diarrheal deaths [3]. Rotavirus is also the prominent cause of acute, moderate to severe diarrhea among children under five years presenting at healthcare facilities in Africa [4], [5]. In Ghana, diarrhea accounts for about 28% of hospital admissions for children under five years [6].

A variety of interventions to manage diarrhea and prevent mortality are available and effective, including micronutrient supplementation and oral rehydration therapy [7], and improvements in sanitation and water quality. However, coverage of micronutrient supplementation and oral rehydration remains low in Africa, and the benefits of infrastructure improvements often accumulate over the longer term [8], limiting the immediate impact on morbidity and mortality [4], [9]. Rotavirus vaccination has been proven to significantly reduce diarrheal hospitalizations [10], [11] and deaths [12], [13]. Based on this evidence, the World Health Organization (WHO) in 2009 recommended that rotavirus vaccination be introduced into the national Expanded Program on Immunization (EPI) [14]. Since introduction, the impact of rotavirus vaccines on reducing disease burden continue to be reported in country-level studies [4], [6], [12]. Gavi also supports rotavirus vaccination, and many countries in the African region, including Ghana, have introduced rotavirus vaccine or are in the process of introduction [15].

WHO has prequalified two rotavirus vaccines, RotaTeq® and Rotarix® [16]. RotaTeq® (RotaTeq is a registered trademark of Merck & Co., Inc.) is a 3-dose oral, live attenuated vaccine; Rotarix® (ROTARIX is a registered trademark of GlaxoSmithKline Biologicals SA, used under license by GlaxoSmithKline Inc.) is a 2-dose oral, live attenuated vaccine. Clinical trials for the RotaTeq vaccine were conducted in Ghana, and then Ghana introduced the Rotarix vaccine in 2012.

Studies have illustrated the economic and health impact of rotavirus disease on households and countries [17], [18], [19], [20], [21], [22]. Further, studies have shown that rotavirus vaccination is cost-effective in children under five years [23], [24], [25], [26], [27], [28], [29], [30]. However, many of these studies assessed the cost-effectiveness of potential rotavirus vaccine introduction programs, with limited evidence on cost-effectiveness of existing programs, especially in countries that are now approaching Gavi transition. While evidence to inform introduction is important, evidence after introduction and as countries bear a larger portion of the cost is also crucial to determine the economic value of these investments given the changing circumstance (e.g. transition).

Ghana’s transition from low to lower-middle income country (LMIC) status in 2010 came with significant reduction in general donor support. Net official development assistance per capita fell by 30% between 2011 and 2013, with donor budget support also declining from about 22% to about 6% over the same period [31]. Donor agencies such as the Danish Development Agency (DANIDA) have either ended or disbursed final budget support while others have indicated plans to withdraw sector budget support. Ghana is also on a path toward Gavi transition, which has been projected to occur in 2022, after which the country will bear the full cost of vaccines. Ghana, and countries which are receiving declining international financial support, still face significant disease burden but will have fewer resources to address these challenges. In this context, a cost-effectiveness analysis (CEA) is an important tool to help policymakers assess the pros and cons of competing priorities, thereby informing the allocation (or reallocation) of limited resources. Unlike previous studies, this study examines the cost-effectiveness of continued rotavirus vaccination during a period in which Ghana is in economic transition and expected to assume financial responsibility for vaccines as Gavi support diminishes.

## 2 Materials and methods

### 2.1 Model and analysis perspective

This analysis utilizes the TRIVAC model, which is a Microsoft Excel® based tool developed by researchers at the London School of Hygiene and Tropical Medicine (LSHTM) to perform CEA for rotavirus, pneumococcal, and *Haemophilus influenzae* type b (Hib) vaccines. Funding for TRIVAC was provided by the Bill and Melinda Gates Foundation through PAHO’s ProVac Initiative and by Gavi’s Hib Initiative. Model inputs include population demographics, disease burden, health system structure, utilization and costs as well as vaccine cost, coverage and efficacy. Table 1, Table 2, Table 3 and the appendix contain key data inputs utilized in the analysis. Additional detail on the TRIVAC model, its structure, methodology and default inputs has been published elsewhere [32].

**Table 1:** Input parameters for estimating disease burden.

Parameter	Estimate	Source
*Annual incidence per 100,000*		
Rotavirus (non-severe)	9290	Assumption, per [35], [36]
Rotavirus (severe)	710	Assumption, per [35], [36]
% Case fatality ratios for severe disease	6.50%	Calibrated to align with [3]

*Disability weights for DALY calculation*		
Rotavirus (non-severe)	0.19	[54]
Rotavirus (severe)	0.25	[54]
Mean duration of illness (days)		
Rotavirus (non-severe)	3	[55]
Rotavirus (severe)	5	[55]
Age distribution of disease cases and deaths		[55]
<3 m:	7.1%	
3–5 m:	17.1%	
6–8 m:	22.9%	
9–11 m:	22.9%	
12–23 m:	28.6%	
24–35 m:	1.4%	
36–47 m:	0.0%	
48–59 m:	0.0%

**Table 2:** Input parameters for estimating health service utilization and costs.

Indicator	Estimate	Source
*Outpatient visits per non-severe episode*	0.45	[37]
*Household cost per outpatient visit*		Assumption, based on [42]
Private Clinic	$1.61	
Private Hospital	$1.69	
Public Health Center	$0.72	
Public District Hospital	$1.10	
Public Regional Hospital	$1.36	
Public Teaching Hospital	$2.75	

*Government cost per outpatient visit*		[40]
Private Clinic	$3.59	
Private Hospital	$3.77	
Public Health Center	$1.61	
Public District Hospital	$2.47	
Public Regional Hospital	$3.05	
Public Teaching Hospital	$6.15	

*Inpatient visits per severe episode*	0.80	Assumption, based on local expert opinion
*Household cost per outpatient visit*		Assumption, based on [42]
Private Hospital	$15.67	
Public District Hospital	$10.17	
Public Regional Hospital	$16.94	
Public Teaching Hospital	$21.74	
*Government cost per outpatient visit*		[40]
Private Hospital	$35.02	
Public District Hospital	$22.71	
Public Regional Hospital	$37.85	
Public Teaching Hospital	$48.57

**Table 3:** Input parameters for estimating health impact of rotavirus vaccination.

Indicator	Estimate (%)	Source
Rotavirus, non-severe		
*1 Primary dose*	32.5	[46]
*2 Primary doses*	65.0	

Rotavirus, severe		
*1 Primary dose*	32.5	[46]
*2 Primary doses*	65.0	
*% Decrease in dose efficacy/year*	54.7	[46]

This analysis examines the impact of rotavirus vaccination on 20 birth cohorts followed from birth to age five. The analysis begins in 2012 and extends through the 2031 birth cohort. The costs and benefits of rotavirus vaccination are compared to no vaccination. Vaccine costs are estimated under two scenarios: Scenario 1 uses only the portion of the per dose vaccine price paid by Ghana for the 20-year period, while Scenario 2 uses the per dose vaccine cost borne by Ghana and Gavi. The first scenario allows us to assess the cost-effectiveness of rotavirus vaccination from Ghana’s perspective, as the country transitions away from Gavi support. The second scenario examines the same transition accounting for the full price of the vaccine. This is a clearer representation of the cost-effectiveness of the vaccination program after the Gavi transition occured since it presents the full price Ghana will bear after it transitions. The analysis is conducted from both the healthcare system (i.e. government) and societal perspectives. Costs and health outcomes are discounted at 3% per year. Costs are reported in 2015 US$.

### 2.2 Demographic and disease burden data

Basic demographic data including live births, infant mortality rate, under-five mortality rates and life expectancy at birth were obtained from the United Nations Population Division World Population Prospects 2015 Revision [33]. The incidence of Rotavirus gastroenteritis (RVGE) is not available at a global level or in Ghana so we assume 10,000 per 100,000 cases in children under five years based on the pooled estimate from a global meta-analysis [34]. These cases are then differentiated by severity, with those with a Vesikari score of 15 or higher designated as severe. According to a study conducted by the Navrongo Rotavirus Research group [35], 7.1% of rotavirus cases in Ghana had a Vesikari score of 15 or higher and are classified as severe in the model, which we adopt in this model, with the remaining 92.9% constituting non-severe RVGE cases. Annual rotavirus deaths in Ghana without vaccination are estimated at 1770 in the model to approximately align with 2013 WHO and CDC estimates for Ghana [36]. Finally, disability weights for DALY calculations come from the 2013 Global Burden of disease study [37]. Table 1 presents the key input parameters used in estimating disease burden.

### 2.3 Health system, utilization and cost data

The Ghanaian (public) health care system is structured along the lines of decentralized governance system, the three main levels i.e. the national, regional, and district (which incorporates the sub-district) levels. At each level, there are health facilities that provide services and serve as referral points to other lower level facilities. At the national level, there are four tertiary (also known as teaching) hospitals, which form the apex of the referral system. At the regional levels, there are regional/secondary hospitals which serve as referral facilities to lower level hospitals. District hospitals are primary care hospitals, which also serve as referral points for clinics, health centers, and community-based health planning and services compounds. There are also private and mission health facilities (operated by religious organizations), which deliver health care across the country.

The National Health Insurance Scheme (NHIS), which started operation in 2004, covers all types of providers nationally and reimburses providers for the services provided to the clients of the scheme. The health care system is managed by the Ministry of Health, with assistance from other agencies including Ghana Health Service and Teaching Hospitals (which provide services) and the National Health Insurance Authority (which funds services under the NHIS).

For this study, data on access to care is based on the percentage of children who received advice or treatment from a health facility or provider when experiencing diarrhea as specified by the 2014 Demographic and Health Survey [38]. We apply this percentage (45%) to non-severe cases, while the percentage of severe cases (80%) seeking care is based on local expert opinion. The model assumes that patients access the health system through public or private providers. Those with non-severe disease seek outpatient care through a private clinic, private hospital, public health center, or a public hospital at one of three levels (district, regional, or teaching/tertiary). Those with severe disease seek care in a private hospital or a public district, regional, or teaching hospital. Outpatient cases are distributed among the six outpatient options with 5% seeking treatment at each of four types (i.e. private clinics, private hospitals, regional hospitals or teaching/tertiary hospitals). The remaining 80% of the population seeking treatment is evenly divided between public health centers and district hospitals. About 15% of inpatient cases seek care in a private hospital, 5% from teaching hospitals, and the remaining 80% is evenly divided between district and regional hospitals. The distribution of treatment is assumed based on local expert opinion.

Provider treatment cost data inputs are taken from Ghana’s NHIS 2015 tariff schedule for the treatment of diarrhea and vomiting and differentiated by level of care [39]. Reimbursement rates for the same type of condition differ across providers based on the provider type and level – i.e. a public hospital receives a different rate from a private hospital for treating the same condition. Similarly, a regional hospital gets reimbursed a higher rate than a district hospital for treating the same condition [40]. The differentiation is an attempt to adequately reimburse providers for actual resources expended in treating clients. These input values were reported in 2015 Ghanaian Cedi and converted to 2015 US$ using mid-year exchange rates [40]. In addition to facility costs, which do not include medicines, one oral rehydration solution (ORS) packet and saline drip are included in the cost of treatment for inpatient care. Only ORS is included for outpatient care. Provider treatment cost inputs range from $1.61 for an outpatient visit at a public health center to $6.15 for an outpatient visit at a public teaching hospital. Government costs of inpatient admissions range from $22.71 at a public district hospital to $48.57 at a public teaching hospital. NHIS tariffs for private care are slightly lower than the most expensive public tariffs. For household costs, we assume that the household share of total rotavirus costs is equivalent to the out-of-pocket cost share of Ghana’s total health expenditure. We obtain these national level metrics from the WHO Global Health Expenditure Database [41] and then estimate the unknown household costs, here represented on the left side of the relationship as Private Health Expenditure:

Private Health Expenditure=Total Health Expenditure-General Government Health Expenditure.

Knowing the relative size of these components allows us to estimate household costs based on our knowledge of government rotavirus treatment costs. This estimate yields household costs that range from $0.72 to $2.75 based on the level of the facility in which care is provided. For comparison, median household costs associated with diarrhea treatment in The Gambia, Mali, and Kenya have been reported as $2.63, $4.11, and $6.24, respectively [20], indicating that our estimate of household costs is relatively conservative. Table 2 shows input parameters for estimating service utilization and costs. This study includes only direct treatment costs.

### 2.4 Vaccine costs, coverage and efficacy estimates

Vaccine cost per dose includes the vaccine price, international handling and international delivery fees. With respect to vaccine price, Ghana entered Gavi’s “accelerated transition” phase in 2017 and is expected to graduate from Gavi support in 2022 [42]. At the time of vaccine introduction in 2012, Ghana paid $0.20 per dose based on the Gavi co-financing policy [43], a price that increases to $2.02 in 2022 and is stable thereafter. See Table 1A in the appendix for additional detail. A vaccine wastage rate of 5% is included. The incremental system cost per dose ($1.30) is derived from a published source [44]. Start-up costs, including cold chain, are not included as these are sunk costs and the vaccination program is already functioning.

The Rotarix vaccine being used in Ghana is a two-dose vaccine administered within the current EPI infant vaccination schedule. Coverage levels of DTP1 and DTP2 are used as proxies for rotavirus vaccine coverage. Coverage of DTP1 and DTP3 were accessed through WHO’s immunization coverage database, and DTP2 values were interpolated from DTP1 and DTP3 [45]. Relative coverage is adjusted to account for those at the highest risk of rotavirus mortality also being less likely to receive the vaccine. A full description of this parameter is available elsewhere, but in this case this parameter serves to reduce actual effectiveness by a factor of 0.9 [33].

Rotavirus vaccination has been shown to be efficacious against disease [46]. We use the “first year of life” RotaTeq trial estimate of efficacy (65%) and conservatively assume the efficacy of a single dose is half that of the full course. The protective effect of vaccination may decline over time so we include 55% annual waning to account for the waning efficacy documented between year 1 and year 2 in the RotaTeq trial in Ghana. Efficacy estimates of rotavirus vaccination are presented in Table 3.

To explore the impact of parameter uncertainty on the incremental cost-effectiveness ratio (ICER) of rotavirus vaccination, we performed a one-way sensitivity analysis for key parameters for both scenario 1 and 2 from the societal perspective. Variables include incidence of disease, share of severe disease, case fatality ratio, vaccine efficacy, relative coverage, delivery cost per dose, inpatient and outpatient treatment costs, and utilization rates for severe cases.

## 3 Results

### 3.1 Health impact of vaccination

Table 4 shows the health impact of vaccination comparing the continued vaccination program to a scenario without the vaccination program. Over the 2012 to 2031 period, it is projected that more than 16 million children under five years will be immunized, averting more than 2.2 million cases, more than 255,000 DALYs, and 8900 deaths. Vaccination is projected to lead to more than 250,000 healthy life years gained over the 20-year period.

**Table 4:** Health impact of rotavirus vaccination, 2012–2031.

Indicator*	No vaccine	RV vaccine	Averted
Number of immunized children	–	16,390,860	–
Total cases	6,379,874	4,145,727	2,234,147
Non-severe cases	5,926,903	3,851,380	2,075,523
Severe cases	452,971	294,347	158,624
Total deaths	25,568	16,622	8946
Total DALYs	728,840	4,737,100	255,140
Years of Life Lost (YLLs – DALYs due to mortality)	718,150	466,750	251,400
Years Lived with Disability (YLDs – DALYs due to morbidity)	10,690	6950	3740
Total life years gained	–	251,399	–

* Future health benefits are discounted at 3% per year.

### 3.2 Cost and cost-effectiveness of vaccination

Table 5 shows that, for both the “Ghana only” (Scenario 1) and “total price” (Scenario 2) scenarios, continued vaccination is expected to avert approximately $6.3 million and $9.2 million from the health system and societal perspective, respectively. Table 2A in the appendix includes further detail. The costs averted due to vaccination are higher from the societal perspective due to the inclusion of averted household out-of-pocket costs. The net cost of the vaccination program over the 2012 to 2031 period is approximately $64 million from the health system and $61 million from the societal perspective if only Ghana’s portion of per vaccine dose price are considered. Total net costs of the vaccination program are higher if both Ghana’s and Gavi’s portions of the vaccine price are used – approximately $88 million and $85 million from health system and societal perspectives, respectively.

**Table 5:** Incremental cost of rotavirus vaccination, 2012–2031 (millions $).

Indicator**	Scenario 1*		Scenario 2^	
	Government perspective	Societal perspective	Government perspective	Societal perspective
Total vaccine and program costs	70.00	70.00	93.99	93.99
Treatment cost savings	6.34	9.18	6.34	9.18
Total net costs	63.66	60.82	87.65	84.82

Notes:

* Scenario 1 represents the price paid by Ghana per dose.

^ Scenario 2 represents the total price per dose of vaccine, which includes the price paid by GAVI and the price paid by Ghana.

** Future costs are discounted at 3% per year.

Table 6 shows that the ICERs of rotavirus vaccination are $249 and $238 per DALY averted from health system and societal perspectives, respectively (Scenario 1), and $344 and $332 per DALY averted from health system and societal perspectives, respectively (Scenario 2). Furthermore, the incremental cost per fully immunized child is between $4.27 and $5.73 depending on whether we account for Ghana’s share of the vaccine price or also include Gavi’s share. Further, rotavirus vaccination averts one case at between $27 and $38 depending on the perspective and scenario used. Again, rotavirus vaccination averts one death at $7115 and $6798 from health system and societal perspectives, respectively (Scenario 1), and $9798 and $9481, respectively for Scenario 2.

**Table 6:** Cost-effectiveness of rotavirus vaccination, 2012-2031.

Indicator	Scenario 1*		Scenario 2^	
	Government perspective	Societal perspective	Government perspective	Societal perspective
Cost per immunized child ($)	4.27	4.27	5.73	5.73
Cost per case averted ($)	28	27	39	38
Cost per DALY averted ($)	249	238	344	332
Cost per death averted ($)	7115	6798	9798	9481
Cost per life-year gained ($)	253	242	349	337

Notes:

* Scenario 1 represents the price paid by Ghana per dose.

^ Scenario 2 represents the total price per dose of vaccine, which includes the price paid by GAVI and the price paid by Ghana.

### 3.3 Sensitivity analysis

The base case ICERs from a societal perspective range from $238 per DALY averted to $332 per DALY averted in scenario 1 and 2, respectively. Under one-way sensitivity analyses of the most influential factors, results vary from $135 to $610 per DALY averted, all well below the highly cost-effective threshold of $1328, the GDP per capita for Ghana in 2015 [47]. In both scenarios, the most influential drivers of the cost-effectiveness ratio are the case-fatality ratio, share of severe disease, and delivery cost per dose. Figs. 1 and 2 are available in the appendix.

## 4 Discussion

This analysis illustrates that the continuation of Ghana’s rotavirus vaccination program substantially reduces child mortality and illness due to rotavirus. It is also expected to be highly cost-effective over the next two decades, even during the period when support from Gavi declines and Ghana assumes full economic responsibility for the vaccination program.

The results obtained in this analysis are broadly consistent with prior studies of rotavirus vaccination programs in developing countries, which demonstrate that rotavirus vaccination is both effective and highly cost-effective [4], [26], [48], [49], [50], [51] in reducing the burden of diarrhea in children under five years. Other work in Ghana also finds rotavirus vaccination to be highly cost-effective [23]. Our analysis suggests that rotavirus vaccination would reduce rotavirus mortality, disease and deaths by 35%. In contrast, a recent analysis of vaccine impact and effectiveness in Ghana reported a 49% reduction in hospitalizations over the first three years after vaccine introduction, highlighting the conservative nature of the results we report [52].

This analysis differs from prior rotavirus cost-effectiveness work in critical ways. In addition to presenting the cost-effectiveness of rotavirus vaccination using the current vaccine price per dose paid by Ghana, the current study also reports ICERs for a scenario representative of Ghana assuming full financial responsibility of vaccines. This finding is crucial for other countries in economic transition and facing reduced external support for health. Thus, the analysis shows that even with the economic challenges that transition economies face (or will likely face), investment in rotavirus vaccination is economically justified, as it produces substantial health returns through a highly cost-effective intervention. This finding provides much needed evidence to policymakers and other stakeholders as it demonstrates the continued cost-effectiveness of rotavirus vaccination in Gavi transition economies.

The ICERs reported here, though still highly cost-effective, are somewhat higher than often reported in the literature. In comparison to similar work on the CEA of rotavirus vaccination in Ghana, many of the inputs used in this study are conservative and this is reflected in our results [23]. The current analysis assumes lower vaccine efficacy and uses updated estimates of rotavirus mortality in Ghana which are much lower than those reported previously. We also utilize lower costs for rotavirus treatment relative to costs collected elsewhere in the region, and our case fatality rate for severe cases is dramatically lower.^2^ In addition, this analysis excludes herd effects and the substantial benefits that may accrue to unvaccinated populations. Finally, it is important to note that the present analysis examines the continuation of a successful rotavirus vaccination program in the context of declining child mortality rather than the introduction of a new program. As rotavirus mortality rates decline due to increased vaccination and a broader decline in diarrhea and child mortality, it is expected that ICERs, particularly the cost per death averted, would rise. In such an instance, it is reasonable to argue that mortality may not necessarily be the best measure to judge the economic value of interventions. Rather morbidity indicators such as the number of severe and non-severe cases averted become crucial, and large, measures of impact. This argument has been made in the case of malaria control, as recent studies have reported higher ICERs in Ghana [53], [54].

Our analysis shows that over a 20-year period, total vaccine and program costs could amount to approximately $70 million under Scenario 1 and $94 million under Scenario 2, implying $3.5 million and approximately $4.7 million per annum. The estimated annual vaccine and program costs account for between 1% and 1.4% of Ghana MoH’s 2015 expenditure on goods and services, pointing to the affordability of rotavirus vaccination.

Economic analyses projecting the costs and benefits of programs can be effective tools to aid decision-making. However, the limitations of models and the data they rely on must also be considered. Substantial efforts were made to ensure the model was populated with the best available data, and the results have been further examined through a sensitivity analysis. A few areas with limited data should be noted. First, the burden of rotavirus disease and the distribution of severe and non-severe cases are not completely understood. Estimates in the model are well-aligned with other work, but remain uncertain. Our data for cost of care reflects recent NHIS tariffs in Ghana. Tariffs may not be a perfect representation of the costs of care, but we believe it to be a fair representation as it corresponds to other estimates based on data collected in Northern Ghana [17] and the international literature [21]. Finally, our understanding of providers visited for rotavirus treatment (i.e. public vs. private, district hospital vs. regional hospital) and the rate at which treatment is sought for severe disease are based on local expert opinion, which may not perfectly reflect treatment seeking behavior across the country. While we would be remiss not to mention these caveats, this analysis has been conducted with conservative assumptions and our sensitivity analysis has explored the areas where changes in input parameters are most likely to affect our results. Even under our conservative assumptions, we find rotavirus vaccination to be highly cost-effective, and this result is consistent across a range of plausible scenarios considered. As Ghana transitions from Gavi support and other international financing mechanisms and assumes full responsibility for immunization and other aspects of health care, it is important to note that continued vaccination represents an efficient way of reducing diarrhea deaths in children under five years.

In conclusion, rotavirus vaccination is highly cost-effective and will continue to yield substantial health and economic impact for the Ghanaian people.

## Conflict of interest

The authors have no conflicts to declare.

## Funding

This work was supported by the Bill & Melinda Gates Foundation, Seattle, WA [Grant No. OPP1147721].

## Appendix A Supplementary material

Supplementary Table 1A

Supplementary Table 2A

Supplementary Fig. 1A

Supplementary Fig. 2A

## Footnotes

2 Note that Abbot et al. do not report a CFR, but they do report the birth cohort and the proportion of children treated as inpatients and outpatients. Assuming all inpatient and outpatient cases are at equal risk of mortality yields a CFR of 2.76%. Assuming only inpatient cases are at risk of mortality yields as CFR of 36.2%. The current analysis assumes an inpatient CFR of 6.5%.
