# Supplementary material for: Cost-effectiveness of rotavirus vaccination in Ghana: Examining impacts from 2012 to 2031
Source: Vaccine. 2018 Nov 12;36(47):7215–21. doi: 10.1016/j.vaccine.2017.11.080 (PMC6238184; doi:10.1016/j.vaccine.2017.11.080)
Supplement: Supplementary Table 1A [file mmc1.docx]

Appendix

Table 1A: Rotavirus vaccine dose price projection ($)

| Year | Scenario 1* | Scenario2^ | Source | | |
| --- | --- | --- | --- | --- | --- |
| 2012 | 0.20 | 2.55 | Estimates based on [43] and  vaccine price assumptions | | |
| 2013 | 0.23 | 2.55 |  |  |  |
| 2014 | 0.26 | 2.57 |  |  |  |
| 2015 | 0.30 | 2.28 |  |  |  |
| 2016 | 0.57 | 2.05 |  |  |  |
| 2017 | 0.53 | 2.19 |  |  |  |
| 2018 | 0.83 | 2.02 |  |  |  |
| 2019 | 1.13 | 2.02 |  |  |  |
| 2020 | 1.42 | 2.02 |  |  |  |
| 2021 | 1.72 | 2.02 |  |  |  |
| 2022 | 2.02 | 2.02 |  |  |  |
| 2023 | 2.02 | 2.02 |  |  |  |
| 2024 | 2.02 | 2.02 |  |  |  |
| 2025 | 2.02 | 2.02 |  |  |  |
| 2026 | 2.02 | 2.02 |  |  |  |
| 2027 | 2.02 | 2.02 |  |  |  |
| 2028 | 2.02 | 2.02 |  |  |  |
| 2029 | 2.02 | 2.02 |  |  |  |
| 2030 | 2.02 | 2.02 |  |  |  |
| 2031 | 2.02 | 2.02 |  |  |  |
| Incremental  system cost per dose | 1.30 | | |  | [44] |

Notes: *Scenario 1 represents the price paid by Ghana per dose. ^Scenario 2 represents the total price per dose of vaccine, which includes the price paid by GAVI and the price paid by Ghana.
