# Supplementary material for: Cost-effectiveness of rotavirus vaccination in Ghana: Examining impacts from 2012 to 2031
Source: Vaccine. 2018 Nov 12;36(47):7215–21. doi: 10.1016/j.vaccine.2017.11.080 (PMC6238184; doi:10.1016/j.vaccine.2017.11.080)
Supplement: Supplementary Table 2A [file mmc2.docx]

| Table 2A: Health service costs averted ($) |  | | |
| --- | --- | --- | --- |
| Indicator | No vaccine  (status quo) | RV Vaccine | Averted |
| Total health service costs  (government perspective) | 18,108,316 | 11,767,025 | 6,341,742 |
| Total outpatient visit costs | 6,546,501 | 4,254,004 | 2,292,948 |
| Total inpatient visit costs | 11,561,814 | 7,513,021 | 4,048,794 |
| Total health service costs  (societal perspective) | 26,206,906 | 17,029,597 | 9,177,310 |
| Total outpatient visit costs | 9,469,807 | 6,153,607 | 3,316,200 |
| Total inpatient visit costs | 16,737,099 | 10,875,990 | 5,861,110 |
