# Supplementary figures and images for: Cost-effectiveness of rotavirus vaccination in Ghana: Examining impacts from 2012 to 2031
Source: Vaccine. 2018 Nov 12;36(47):7215–21. doi: 10.1016/j.vaccine.2017.11.080 (PMC6238184; doi:10.1016/j.vaccine.2017.11.080)

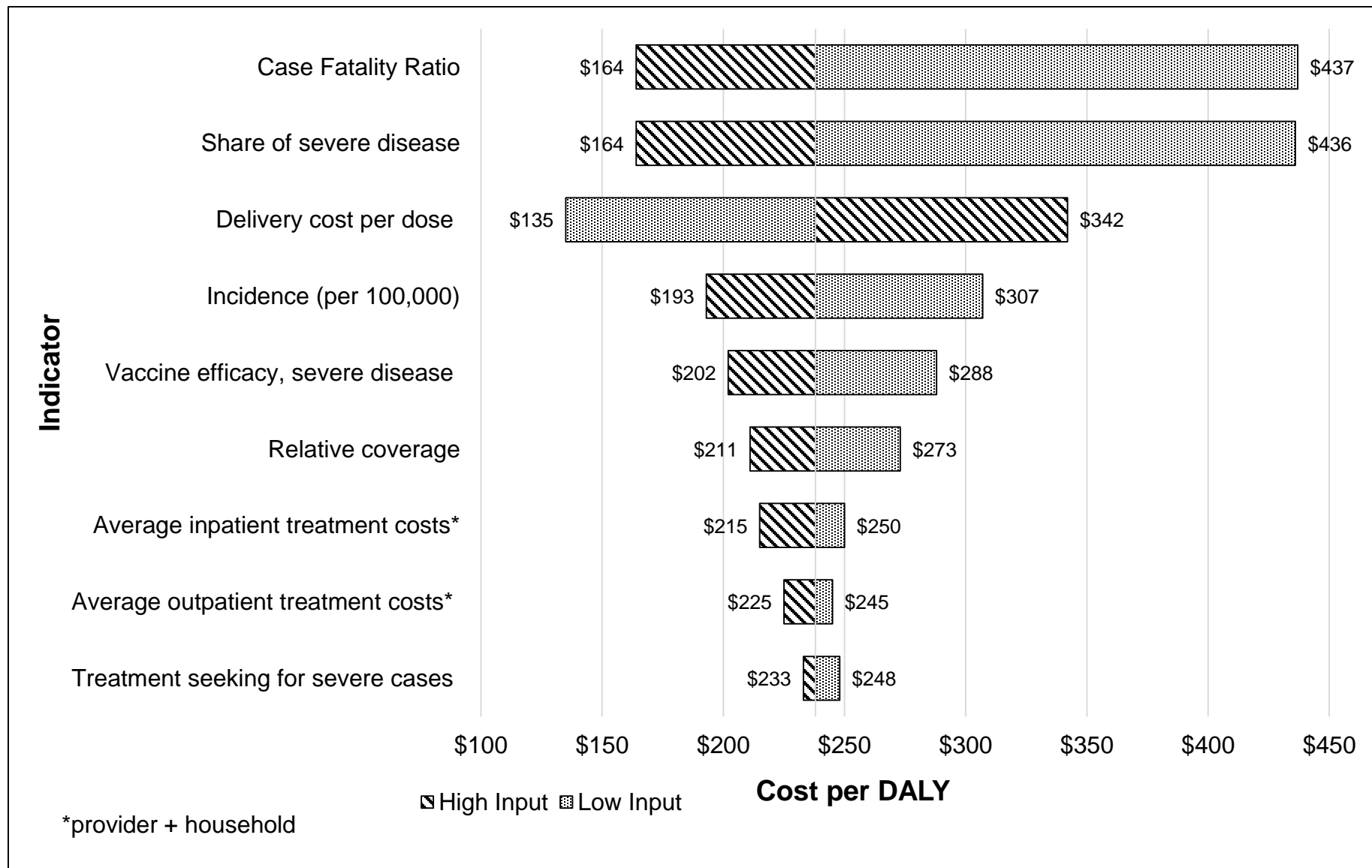

Supplement: Supplementary Fig. 1A [file mmc3.pdf]

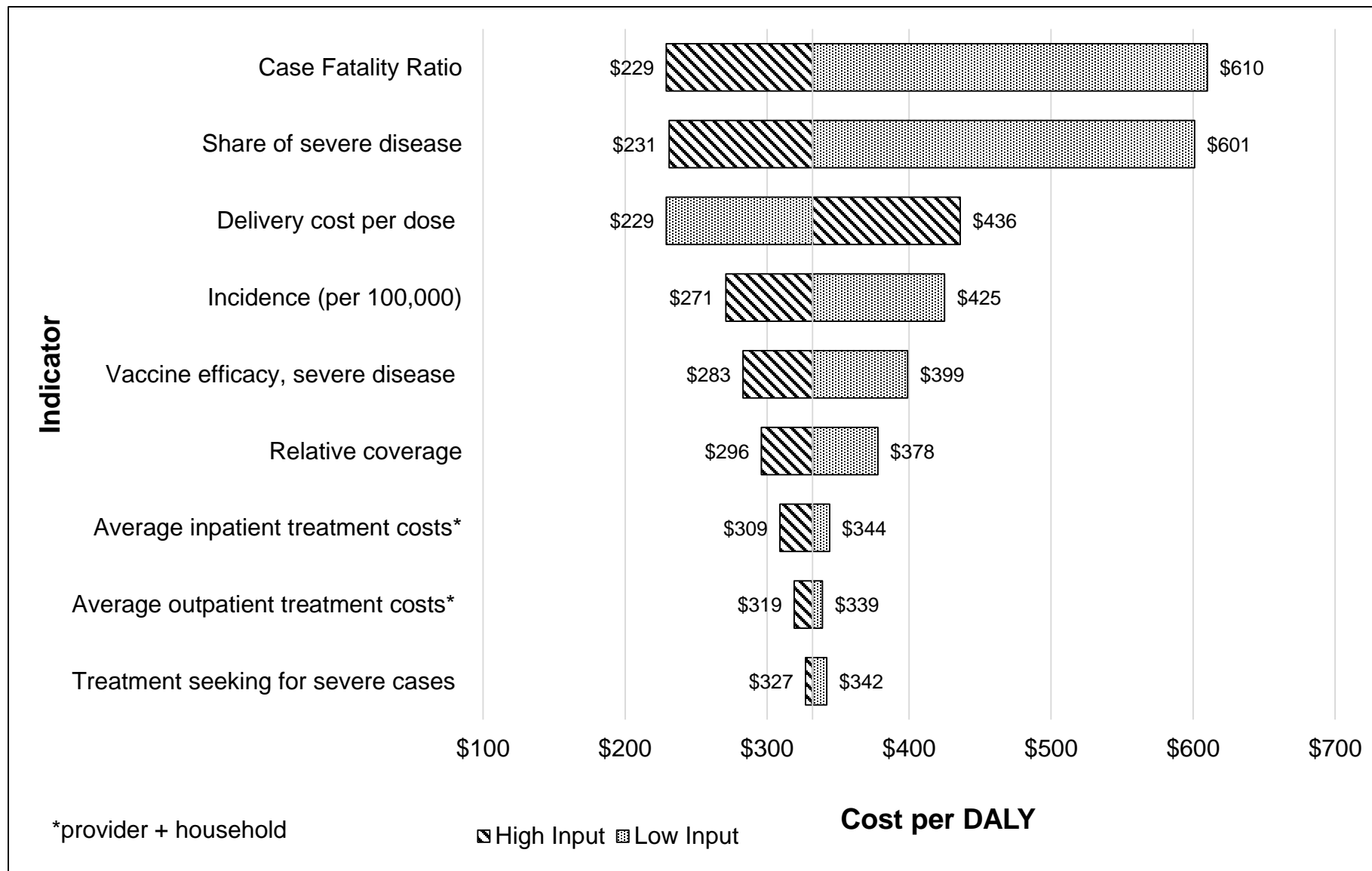

Supplement: Supplementary Fig. 2A [file mmc4.pdf]
